# Gnotobiotic growth and phosphorus limitation of *Arabidopsis thaliana* and co-occurring microbes on phosphated iron oxides

**DOI:** 10.1007/s10534-025-00767-6

**Published:** 2025-11-27

**Authors:** Amanda M. Mackie, Christopher J. Schuler, Darcy L. McRose

**Affiliations:** Department of Civil and Environmental Engineering, Massachusetts Institute of Technology, Cambridge, MA 02139 USA

**Keywords:** Phosphorus, Iron, Arabidopsis thaliana, Rhizosphere, Plant-microbe, Mineral adsorption

## Abstract

The macronutrient phosphorus is vital for sustaining cellular processes in all life forms. Due to its frequent adsorption on iron minerals, phosphorus bioavailability is low in many soils. While the abiotic adsorption of phosphate on iron minerals has been well studied, the direct effects of this process on bioavailability to plants and microbes has not been thoroughly investigated in a simplified laboratory system. We developed a hydroponic growth system that uses hydrous ferric oxide (HFO) to induce phosphorus limitation and can enable both plant and microbial cultivation as well as gnotobiotic co-culture. We demonstrate that this system can be used for phosphorus-limited growth of the model plant *Arabidopsis thaliana* as well as two root-associated bacterial isolates (from the genera *Rhizobium* and *Pseudomonas*). Elemental analysis of phosphorus and iron concentration in *A. thaliana* shoots reveals that the addition of increasing amounts of HFO leads to a progressive decrease in phosphorus concentration but does not affect iron quotas. We also report that phosphorus concentrations in both bacterial isolates decrease when cultivated in media supplemented with HFO. We further show that *A. thaliana* can be co-cultured with a *Rhizobium* isolate in our phosphorus-limited hydroponic system with bacteria relying on plant photosynthate as their sole carbon source. Our work provides a controlled demonstration of the effects of mineral adsorption on phosphorus bioavailability and a tool for further investigation of how plants and microbes access phosphorus in the environment.

**Supplementary Information:**

The online version contains supplementary material available at 10.1007/s10534-025-00767-6.

## Introduction

Phosphorus (P) is an essential nutrient that is required by all forms of life, but it is often unavailable for uptake by plants and microbes in soils. Access to phosphorus constrains primary production in many natural and agricultural environments, and understanding biological responses to this limitation is crucial for both studying and sustainably managing these systems (Van Cappellen and Ingall 1996; Du et al. 2020; Brownlie et al. 2021; Singh et al. 2019; Singh Rawat et al. 2023; Lyu et al. 2021). While many studies have addressed phosphorus stress physiology in model plants and microbes, these investigations have typically done so by simply lowering phosphorus concentrations in the growth media (Danhorn et al. 2004; Zhang et al. 2022; Romano et al. 2015; Ding et al. 2021; Demirer et al. 2023; Malhotra et al. 2018; Santos-Beneit 2015; Conaway et al. 2024). This method has the advantage of offering precise control of soluble phosphorus concentrations but provides more limited insight into the mechanisms through which plants and microbes access unavailable phosphorus in terrestrial environments, where it can be unevenly distributed and adsorbed to soil minerals. The general heterogeneity of soils has long been a barrier to more environmentally realistic studies. However, recent successes in developing laboratory systems that mimic the physical structure of soils have made this task more tractable and highlighted the need for experimental systems of intermediate complexity that incorporate not only the physical but also the chemical features of the soil environment.

The problem of access to phosphorus in soils is primarily one of chemical speciation and frequently involves the intertwined chemistries of iron (Fe) and phosphorus. Although bulk phosphorus concentrations in natural soils can be in the millimolar range, forms of bioavailable phosphorus (PO_4_^3−^, HPO_4_^2−^ and H_2_PO_4_^−^) are often only in the micromolar range, limiting growth (Hayat et al. 2010; Doydora et al. 2020; Du et al. 2020; Zhang et al. 2022). A common mechanism by which phosphorus becomes inaccessible is through adsorption to minerals such as Fe(III) (oxyhyd)roxides (Borch and Fendorf 2008; Stumm and Sulzberger 1992). Numerous lab-based abiotic studies have probed the adsorption of phosphate to mineral surfaces, investigating kinetics and isotherms for the process across experimental conditions [see (Li et al. 2016) and references therein]. In addition, several field-based investigations have shown that the presence of iron oxides can limit phosphorus uptake by plants and microbes (Gross et al. 2018; Emsens et al. 2017; Giesler et al. 2002; Li et al. 2025; Chacon et al. 2006). Flow through bioreactors (Borch and Fendorf 2008) and *in situ* chemical mapping (Li et al. 2025; Brodersen et al. 2017) studies have provided further evidence that iron adsorption constrains phosphorus mobility. Despite the abundance of evidence for the control of P bioavailability by Fe mineral adsorption, to our knowledge, this mechanism has yet to be tested directly in simplified laboratory experiments where plants, microbes, and minerals are fully defined.

In the last decade, there have been numerous advances in cultivation methods that have allowed for more realistic but still controlled rhizosphere studies in intermediate complexity environments. Devices like the EcoFab (Gao et al. 2018; Zhalnina et al. 2018) and Glo-Roots systems (Rellán-Álvarez et al. 2015; LaRue et al. 2022) have been used to promote flat root growth morphologies, making it feasible to conduct high throughput studies of root phenotypes. Microfluidics has been an especially popular approach, used successfully in the EcoFab as well as devices such as the *Arabidopsis* Root Microbiome Microfluidic Device [ARMM, (Conway et al. 2024)], Tracking Root Interactions System [TRIS, (Massalha et al. 2017)], and RootChip (Grossmann et al. 2011) to enable metabolomic studies of root exudates as well as imaging of bacterial root colonization (Nezhad 2014). Additionally, there have been a number of efforts to develop optically transparent soils and scaffolds to help faciliate imaging (Sharma et al. 2020; Smercina et al. 2022; Quinn et al. 2025). Other researchers have used glass beads (Boiteau et al. 2021) or lithography techniques (Bhattacharjee et al. 2024) to mimic soil structure while still allowing for plant and microbial growth in chemically defined media.

These recent efforts have made great strides in replicating the physical structure of soils. However, there have been fewer attempts to mimic the chemistry of soils in more simplified systems. One notable exception is work by (Hanlon et al. 2018), who used a combination of phosphate-doped aluminum oxides and gel media to provide a buffered phosphate release system for plant growth. This study focused on plants and did not address microbial growth or root colonization. Nonetheless, the authors document large differences in phosphorus stress root phenotypes and tissue quotas between their study and other studies conducted using soluble P, underscoring the need for more realistic replication of both physical and chemical processes in soils. Given the previous success of work buffering phosphorus with aluminum oxides and the fact that the abiotic process of phosphorus adsorption to iron minerals is well understood (Penn et al. 2022; Borch and Fendorf 2008), we sought to incorporate this knowledge into laboratory methods that directly test the bioavailability of phosphate adsorbed to hydrous ferric oxide (HFO), an iron form found ubiquitously in soils.

The goal of this study was to provide a proof-of-concept demonstration of the effects of HFO on phosphorus bioavailability to plants and soil bacteria and to develop tools that can be used to further investigate this process in controlled laboratory systems. Using a combination of elemental biomass quotas and growth assays, we show that increasing HFO additions induce progressive phosphorus limitation in the well-studied model plant *Arabidopsis thaliana* (*A. thaliana*) as well as several *A. thaliana* root-derived bacterial isolates: *Rhizobium* Root 491 and *Pseudomonas* Root 71 (Bai et al. 2015). Co-culture of *A. thaliana* and *Rhizobium* Root 491 on HFO amended media further demonstrates successful root colonization, bacterial growth, and the persistence of phosphorus limitation in co-cultured plants. Overall, our work shows that HFO does indeed limit P bioavailability and provides an experimental system for more environmentally realistic explorations of plant and microbial adaptations to unavailable phosphorus.

## Materials and methods

### Abiotic phosphate adsorption experiments

To mimic plant growth conditions, abiotic experiments were performed in modified 0.5 × Murashige and Skoog (MS) medium at pH 5.5 containing 500 μM phosphorus (132.6 $\mu \text{M }{\mathrm{K}}_{2}{\mathrm{HPO}}_{4}\text{ and }367.4 \mu \text{M K}{\mathrm{H}}_{2}{\mathrm{PO}}_{4}$), 30 mM nitrogen (9.74 mM $${\mathrm{KNO}}_{\begin{array}{c}3\\ \end{array}}$$ and 10.13 mM $${\mathrm{NH}}_{4}{\mathrm{NO}}_{\begin{array}{c}3\\ \end{array}}$$), 50 μM boric acid, 0.73 mM ${\mathrm{MgSO}}_{4}\bullet 7{\mathrm{H}}_{2}\mathrm{O}$, 1.5 mM ${\mathrm{CaCl}}_{2}\bullet 2{\mathrm{H}}_{2}\mathrm{O}$, 5 mM MES buffer, and MS trace metals with or without Fe(III)- EDTA (50 μM ${\mathrm{FeCl}}_{3}\bullet {6\mathrm{H}}_{2}\mathrm{O}$ and 100 μM EDTA). Trace metals and EDTA were pre-equilibrated in the growth medium for least 24 h before the start of experiments. Two-line ferrihydrite, subsequently referred to as Hydrous Ferric Oxide (HFO), was synthesized using the method described by (Schwertmann and Cornell 2000). Mineral identity was confirmed by X-ray diffraction analysis (data not shown). Ground HFO was weighed into trace metal free tubes (Avantor, 89049–170) and media was added to a volume of 12–13 mL to achieve desired concentrations ranging from 0.25 mg to 3 mg HFO mL^−1^ media. Tubes were mixed by inverting several times a day. At each sampling time, 850 μL of mixed sample was applied to a cellulose-acetate spin filter (Costar 8161), centrifuged at 12,500 rcf for 3 min, and 750 μL of flow through was transferred to a new trace metal free tube. This was repeated to obtain a total volume of 1.5 mL of filtered sample for each time point. An initial T0 sample was taken before the addition of ground HFO. Samples were stored at 4 °C in the dark until quantification with Inductively Coupled Plasma Mass Spectrometry (ICP-MS), see below.

### Hydroponic plant growth

All *Arabidopsis thaliana* seeds were Columbia wild type (Col-0) sourced from the *Arabidopsis* Biological Resource Center (ABRC stock number CS70000, NASC stock number N70000). We used two sequential methods to ensure seed sterility: chlorine gas and bleach sterilization. For chlorine gas, seeds were surface sterilized in a sealed container in a fume hood for 1 h and vented for 3 h. Chlorine gas was created using 100 mL of bleach (8.25% sodium hypochlorite, NaClO) and 3.3 mL of 32–35% hydrochloric acid (HCl). Following treatments with chlorine gas, seeds were sterilized again in 1 mL of 20% bleach (1.65% sodium hypochlorite, NaClO) and 0.1% Triton-X-100 while shaking gently for 10 min. Seeds were rinsed 6–8 times by letting them settle to the bottom of the tube, removing the bleach solution with a pipet, and adding 1 mL of sterile MilliQ water. Seeds were stratified in sterile MilliQ water in the dark at 4 °C for 3–7 days.

Hydroponic growth methods were adapted from (Voges et al. 2019). Seeds were planted on circular plastic rafts 3 cm in diameter floating on 4 mL of growth medium in sterile 6-well tissue culture plates (Corning, 3516). Rafts were cut out of rigid high temperature PTFE plastic mesh with openings that are 0.0889 by 0.0178 cm (McMaster-Carr) and sterilized by autoclaving. Plants were grown in modified 0.5 × MS media at pH 5.5 as described above. Before planting, HFO was weighed, ground into a fine powder with a mortar and pestle, and sterilized with 70% ethanol overnight. HFO was then centrifuged at 12,500 rcf for 5 min to remove ethanol, washed with 10 mL of sterile MilliQ water, resuspended in 0.5 × MS media to a concentration of 10 mg HFO mL^−1^ in media, and added to 6-well plates with regular mixing of the stock solution to prevent settling. To achieve the desired final concentrations of 1–3 mg mL^−1^ HFO, sterile 0.5 × MS media was added to a final volume of 4 mL per well. For planting, a pipet was used to place 6 clusters of seeds (suspended in sterile MilliQ water) on plastic rafts, each cluster containing 10–15 seeds. Plates were sealed with micropore tape (Nexcare), wrapped in aluminum foil for etiolation to decrease wetting of shoot tissue, and placed in a grow room at 22 °C for 3 days. Tin foil was removed 3 days after planting and plants were grown at 22 °C with 16 h of light and 8 h of dark per day (photon flux of 100 μmol m^−2^ s^-1^ measured with WALZ Universal Light Meter 500). To counter evaporation, media was changed once a week starting 7 days after planting by removing media with a pipet (without disturbing settled HFO mineral) and adding 4 mL of fresh media to each well. Seedlings were grown for 21 days before harvest for ICP-MS analysis, see below.

### Chlorophyll quantification

For chlorophyll quantification, seedlings were harvested 21 days after planting: shoot tissue was cut from the raft, placed in preweighed tubes, freeze dried for 48 h and stored at – 80 °C. Masses were recorded after freeze drying for normalization. Plant tissue was ground using metal balls and a tissue homogenizer (MP Biomedicals, FastPrep-24) and extracted overnight with 1 mL of 95% ethanol. Prior to absorbance measurements, tubes were centrifuged at 12,000 rcf for 10 min and the supernatant was diluted 1:10 in 95% ethanol. Chlorophyll concentrations were determined using the method decribed by (Ritchie 2008) using absorbance readings at 632 nm, 649 nm, 665 nm, and 696 nm taken on a spectrophotometer (VWR, UV-3100PC) with a pathlength of 1 cm.

### Bacterial growth with HFO dialysis bags

Bacterial strains (*Rhizobium* Root 491 and *Pseudomonas* Root 71) from the *A. thaliana* synthetic community were provided as a gift from Paul Schulze-Lefert at At-RSPHERE (Bai et al. 2015). Strains were maintained as glycerol stocks. Cultures were struck onto LB plates and the resulting colonies were inoculated from plates into 10 mL of pH 5.5 defined media containing 16 mM nitrogen (${\mathrm{NH}}_{4}\mathrm{Cl}$), 148 mM carbon from succinic acid (${\mathrm{C}}_{4}{\mathrm{H}}_{4}{\mathrm{Na}}_{2}{\mathrm{O}}_{4}\bullet {6\mathrm{H}}_{2}\mathrm{O}$), 0.41 mM ${\mathrm{MgSO}}_{4}\bullet 7{\mathrm{H}}_{2}\mathrm{O}$, 0.68 mM ${\mathrm{CaCl}}_{2}\bullet 2{\mathrm{H}}_{2}\mathrm{O}$, 25 mM MES buffer, and Aquil trace metals (which contain 100 µM EDTA, Price et al. 1989) supplemented with 10 μM iron (${\mathrm{FeCl}}_{3}\bullet {6\mathrm{H}}_{2}\mathrm{O}$). To induce phosphorus stress, cultures were pre-grown with 50 μM phosphorus (${16.4 \mu \text{M K}}_{2}{\mathrm{HPO}}_{4}\text{ and }33.6 \mu \text{M K}{\mathrm{H}}_{2}{\mathrm{PO}}_{4}$) and diluted 1:50 in this low phosphorus media every ~ 24 h for 2 days. To initiate experiments, bacterial strains were pelleted at 6800 rcf for 10 min, resuspended in media without phosphorus, and inoculated at an ${\mathrm{OD}}_{500}$ (absorbance at 500 nm) of 0.01 into 25 mL of the media containing 500 μM phosphorus. All cultures were grown at 30 °C with continuous shaking at 200 rpm.

Additionally, for phosphorus limitation with iron oxides, 2 mg HFO mL^−1^ was added. To avoid HFO precipitates contributing to OD measurements, HFO was contained within dialysis bags (Supplementary Fig. 1d). To prepare dialysis bags, HFO was weighed, ground into a fine powder with a mortar and pestle, and sterilized with 70% ethanol overnight. HFO was centrifuged at 12,500 rcf for 5 min to remove ethanol, washed with 7 mL of sterile MilliQ water, and resuspended in media containing 500 μM phosphorus to a concentration of 20 mg HFO mL^−1^ media. Dialysis membrane tubing (Thermo Scientific SnakeSkin 3.5 K MWCO, 88242) was cut to a length of 15 cm and one end was tied into a tight knot, 2.5 mL of the HFO stock solution (20 mg HFO mL^−1^ media) was added to the dialysis tube and a tight knot was tied at the other end of the dialysis tubing. Dialysis bags were then added to flasks containing 22.5 mL media, resulting in a final concentration of 2 mg HFO mL^−1^ in 25 mL media. To facilitate adsorption, HFO dialysis bags were incubated at room temperature in the dark with media containing 500 µM phosphorus for 48 h with shaking at least twice per day before bacterial inoculation. Bacterial growth experiments were conducted at 30 °C with continuous shaking at 200 rpm and growth was measured using absorbance at 500 nm (${\mathrm{OD}}_{500}$).

### Bacterial phosphorus concentrations

To determine bacterial P concentrations, cells were filtered onto 0.2 µm polycarbonate filters. Before filtering samples, glass filter rig frits and funnels were acid washed in 10% hydrochloric acid (HCl) and rinsed with salt solution (0.41 mM ${\mathrm{MgSO}}_{4}\bullet 7{\mathrm{H}}_{2}\mathrm{O}$, 0.68 mM ${\mathrm{CaCl}}_{2}\bullet 2{\mathrm{H}}_{2}\mathrm{O}$). Six to twelve mL of bacterial culture was added to filter funnels depending on the density of the culture. To avoid contamination of measurements from surface adhered phosphorus-iron particles, cells were treated with an EDTA-oxalate solution (Tang and Morel 2006). Briefly, 5 mL of EDTA-oxalate solution (100 mM ${\mathrm{Na}}_{2}{\mathrm{C}}_{2}{\mathrm{O}}_{4}$, 50 mM Na_2_EDTA, 0.41 mM ${\mathrm{MgSO}}_{4}\bullet 7{\mathrm{H}}_{2}\mathrm{O}$, 0.68 mM ${\mathrm{CaCl}}_{2}\bullet 2{\mathrm{H}}_{2}\mathrm{O}$) was added to filter funnels, incubated for 5 min, then washed with 10 mL of salt solution (0.41 mM ${\mathrm{MgSO}}_{4}\bullet 7{\mathrm{H}}_{2}\mathrm{O}$, 0.68 mM ${\mathrm{CaCl}}_{2}\bullet 2{\mathrm{H}}_{2}\mathrm{O}$) before filtering samples. Filters were folded and stored at – 20 °C until processing for ICP-MS analysis, see below.

### Inductively coupled plasma mass spectrometry (ICP-MS)

Abiotic samples from HFO incubations with phosphorus media were prepared for ICP-MS by dilution of 35% nitric acid (HNO_3_, trace metal free) to a final concentration of 2% nitric acid. Bacterial filter samples were prepared for ICP-MS by digestion in 5 mL concentrated nitric acid (70% HNO_3_, trace metal free) for 6 h at 90 °C, followed by fivefold dilution with MilliQ water to a final concentration of 14% nitric acid (HNO_3_, trace metal free). Measured concentrations of phosphorus were normalized per cell using a conversion of ${\mathrm{OD}}_{500}$ of 1 being 4.05 × 10^8^ cells mL^−1^ for the *Pseudomonas* Root 71 strain and 7.45 × 10^8^ cells mL^−1^ for the *Rhizobium* Root 491 strain (as determined separately via Colony Forming Unit (CFU) counting). For plant tissues, 21-day-old *A. thaliana* seedlings were harvested by cutting shoots from the plastic rafts and rinsing them with MilliQ water. For each sample, 3 wells were pooled to achieve dry weights of 5–10 mg (Supplementary Fig. 2) and to maintain values above the limit of detection for phosphorus (~ 20 ppb). Roots were excluded from analysis due to contamination from HFO minerals. Shoots were placed into microcentrifuge tubes and dried at 55 °C for 5–7 days until their mass stabilized. Dried shoots were ground with a mortar and pestle, weighed, and digested in 5 mL of concentrated nitric acid (70% HNO_3_, trace metal free) for 6 h at 90 °C. Digested plant tissue samples were diluted fivefold with MilliQ water to a final concentration of 14% nitric acid (HNO_3_, trace metal free). Measured concentrations of phosphorus and iron were normalized by dry plant mass.

All tubes used for sample preparation were trace metal free (Avantor, 89049–170). Additionally, scandium (Inorganic Ventures, AASC1) was used as an internal standard and was spiked into each sample at a final concentration of 200 ppb before running on the ICP-MS (Agilent Technologies 7900 with an integrated autosampler). Counts per second were converted to ppm or μM using standard curves for phosphorus ranging from 0.02 to 100 ppm (made from Thermo Scientific, 013862.AE) and iron ranging from 0.001 to 10 ppm (made from Thermo Scientific, 047282.AP). A Bonferroni-corrected one-tailed t-test was performed using the R function stat_compare_means.

### *A. thaliana*-bacteria co-culture

For co-culture experiments, the *Rhizobium* strain (Root 491) was streaked from a glycerol freezer stock on to LB agar plates and incubated at 30 °C overnight. Bacteria were inoculated from LB plates into 10 mL of tryptic soy broth (TSB) and incubated overnight at 30 °C with continuous shaking at 200 rpm. Four mL of liquid bacteria culture was pelleted at 6,800 rcf for 10 min, washed three times with 10 mL of 0.5 × MS media, and diluted in 0.5 × MS media containing no Fe(III)-EDTA to an ${\mathrm{OD}}_{500}$ of 0.001. Media was removed from 20-day-old *A. thaliana* seedlings and replaced with either sterile 0.5 × MS media (non-colonized wells) or with 0.5 × MS media with bacteria at an ${\mathrm{OD}}_{500}$ of 0.001 (root colonized wells). To determine counts of planktonic cells, media from colonized wells was removed, serially diluted, and plated for CFU counting on LB agar plates. To quantify root-adhered cells, rafts with root-colonized plants were transferred to new plates with sterile 0.5 × MS media, washed three times, sealed with parafilm, and sonicated for 1 min at 40 kHz to release root adhered microbes into the media. Notably, despite sonication, some cells may still remain root adhered and hence this method provides a lower rather than upper bound on total cells on the roots. Following sonication, media was removed, serially diluted, and plated for CFU counting on LB agar plates. To quantify plant tissue P and Fe concentraion, 21-day-old *A. thaliana* seedlings were harvested for ICP-MS, see above.

## Results

### Abiotic phosphorus adsorption to HFO

Abiotic experiments were performed to determine the ideal conditions to achieve phosphorus limitation in plant and microbial growth experiments. For these experiments, we synthesized 2-line ferrihydrite, herein referred to as HFO, as a mimic for a commonly occurring form of iron in soils. We sought to understand the amount of HFO needed to fully deplete soluble phosphorus as well as the speed of phosphorus adsorption, which is important for assessing the amount of time that HFO should be pre-incubated with phosphorus before beginning biological experiments. Half strength modified Murashige and Skoog (MS) medium with 500 $\mu \mathrm{M}$ phosphorus was incubated with various concentrations of ground HFO for 72 h to allow phosphorus to adsorb onto the HFO surface. The concentration of phosphorus remaining in the media (not adsorbed to iron mineral) was determined by removing the HFO via filtration and measuring phosphorus by ICP-MS.

**Fig. 1:**
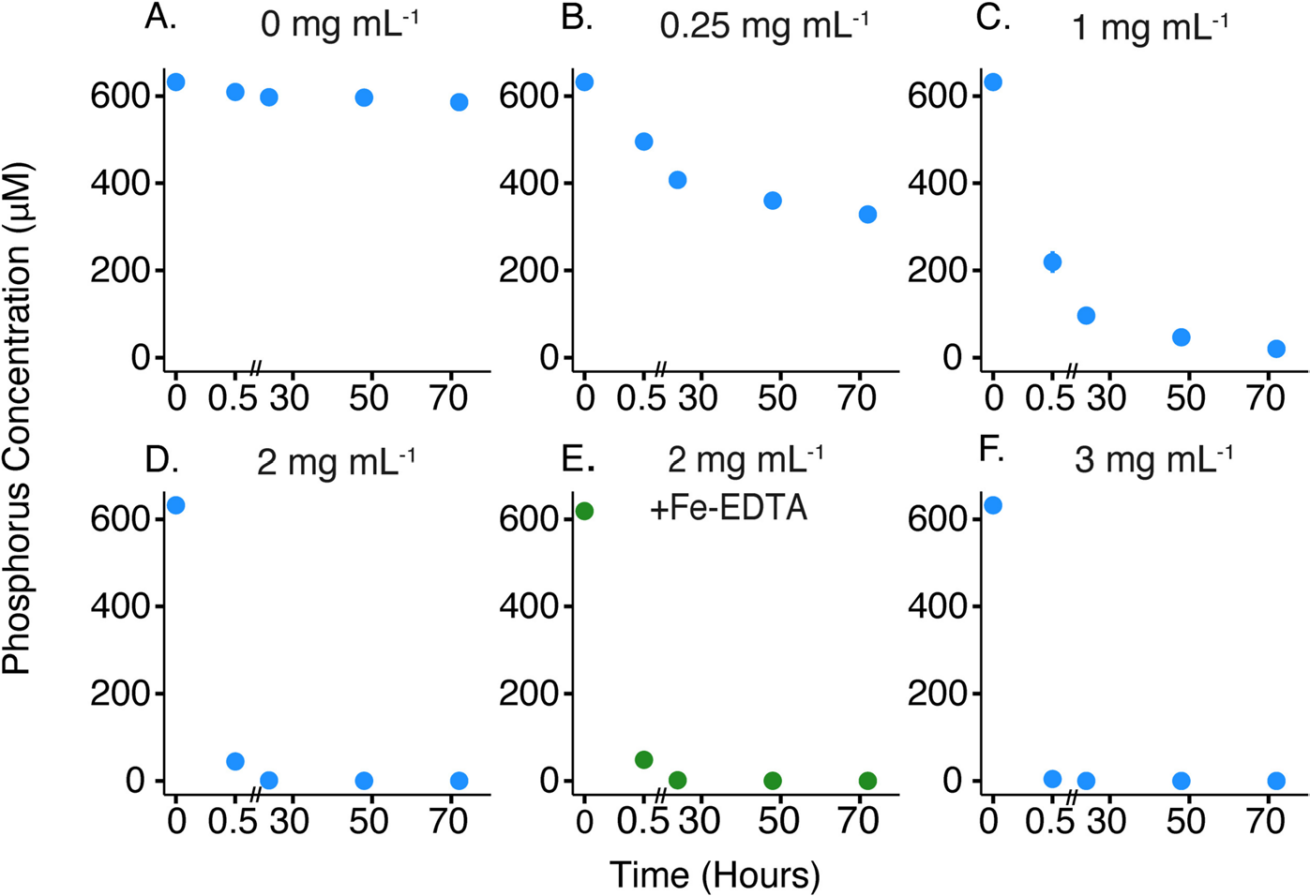
Hydrous ferric oxide scavenges phosphorus. HFO was incubated in 0.5 × MS media with 500 μM phosphorus in all conditions. HFO concentration (mg mL^−1^) and EDTA additions, where present, are indicated above each panel. Half strength MS media contained either no Fe(III)-EDTA (blue, **A**, **B**, **C**, **D**, **F**) or Fe(III)-EDTA (50 μM FeCl_3_ and 100 μM EDTA; green, **E**). Phosphorus concentration in the bulk media (not adsorbed to the HFO mineral) was measured in duplicate. X-axis is zoomed in for 0–0.5 h. Error bars show the average of duplicate replicates ± 1 standard deviation and are sometimes smaller than the symbols

Phosphorus concentrations were stable over time in treatments without HFO (Fig. 1a). For treatments with HFO, within the first 30 min of incubation a substantial portion of the phosphorus in the media was adsorbed to the mineral surface for all HFO concentrations (Fig. 1). Additionally, for HFO concentrations greater than or equal to 1 mg HFO mL^−1^, the concentration of aqueous phosphorus eventually dropped below the limit of detection of (~ 20 ppb), indicating complete adsorption of phosphorus to the mineral surface (Fig. 1c–f). The speed of adsorption was dependent on the HFO concentration with complete adsorption occurring within 72 h for 1 mg mL^−1^, 24 h for 2 mg mL^−1^, and 30 min for 3 mg mL^−1^ (Fig. 1c–f). This result is expected as higher concentrations of HFO provide more mineral surface area for phosphorus adsorption. The 2 mg HFO mL^−1^ condition was also separately incubated with Fe(III)- EDTA (50 $\mu \mathrm{M}$ ${\mathrm{FeCl}}_{3}\bullet {6\mathrm{H}}_{2}\mathrm{O}$ and 100 $\mu \mathrm{M}$ EDTA) to determine if adding EDTA, a chelator often used to control metal bioavailability, would alter phosphorus adsorption to HFO (Fig. 1e). These experiments showed that adding Fe(III)-EDTA did not greatly alter the extent or timing of phosphorus adsorption.

To ensure sufficient phosphorus limitation due to adsorption to HFO, concentrations of 1–3 mg HFO mL^−1^ were used in subsequent biotic experiments and HFO was pre-incubated with phosphate-containing media for at least 48 h to allow full mineral adsorption. For plant growth experiments, this 48-h adsorption period occurred during etiolation (see methods). For bacterial experiments, where growth is much faster, media was pre-incubated with HFO for 48 h before adding cells.

### Phosphorus limitation of *A. thaliana*

**Fig. 2:**
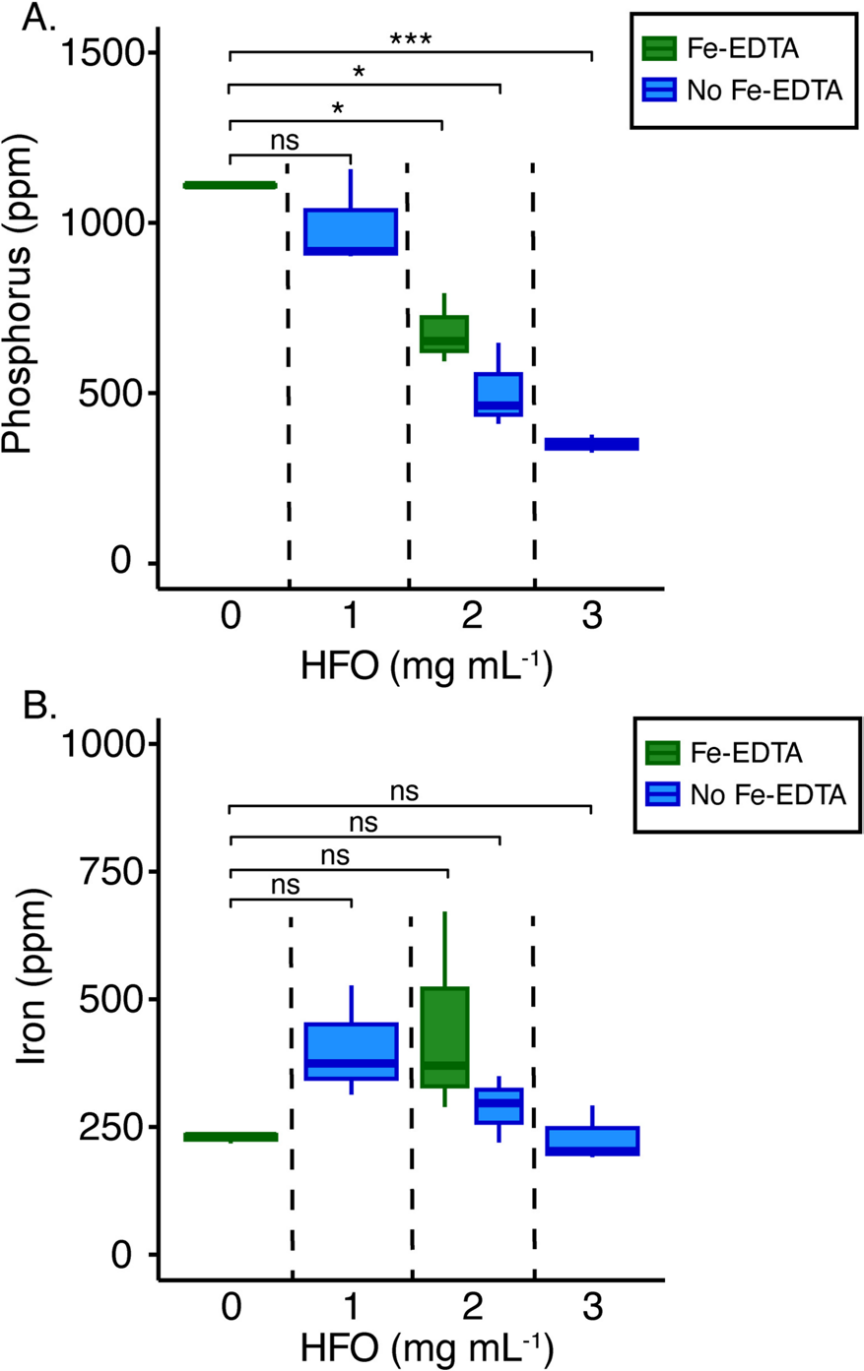
Phosphorus limitation of *A. thaliana* using HFO. *A. thaliana* was grown hydroponically in 0.5 × MS media containing 500 μM phosphorus and various concentrations of HFO. Shoot phosphorus (**A**) and iron (**B**) concentration were quantified. Three biological replicates were analyzed per condition. Fe(III)-EDTA (50 μM FeCl_3_ and 100 μM EDTA) was included for the no HFO condition and for one 2 mg HFO mL^−1^ condition (green). Treatments without Fe(III)-EDTA are indicated in blue. A Bonferroni-corrected one-tailed *t*-test was used where *,**,*** indicate *p*-values less than 0.05, 0.01, 0.001, respectively. No significance is indicated by ns. Box plot shows median and interquartile range

To determine how adsorption to HFO affects phosphorus bioavailability to plants, we grew *A. thaliana* hydroponically (Supplementary Fig. 1a–c) in 0.5 × MS media with 500 $\mu \mathrm{M}$ phosphorus and various concentrations of HFO. After 21 days of growth, phosphorus and iron concentrations in the seedling shoot tissue were measured with ICP-MS (Fig. 2). We found that as the concentration of HFO in the media increased, shoot phosphorus concentration decreased: 2 mg and 3 mg HFO mL^−1^ media resulted in a 54% and 68% decrease, respectively, in phosphorus shoot concentration compared to the condition with no HFO (Fig. 2a). These results were consistent with the abiotic results (Fig. 1), which demonstrated that higher HFO concentrations resulted in lower dissolved phosphorus concentrations in the media and therefore less phosphorus available for uptake by the plant. The addition of the iron chelator EDTA slightly increased shoot phosphorus concentration but did not fully restore it to the phosphorus concentration observed with no HFO present. In contrast to the changes seen in shoot phosphorus, HFO had no effect on shoot iron concentrations (Fig. 2b), suggesting that this hydroponic method induces phosphorus limitation of plants without introducing iron limitation. Notably, while 3 mg mL^−1^ HFO treatments showed the largest decrease in phosphorus shoot concentration, overall plant growth was visibly stunted suggesting that P limitation may be so severe as to make this treatment not experimentally useful. As such, 2 mg HFO mL^−1^ media was used for all following experiments.

### Phosphorus limitation of rhizosphere bacteria

**Fig. 3:**
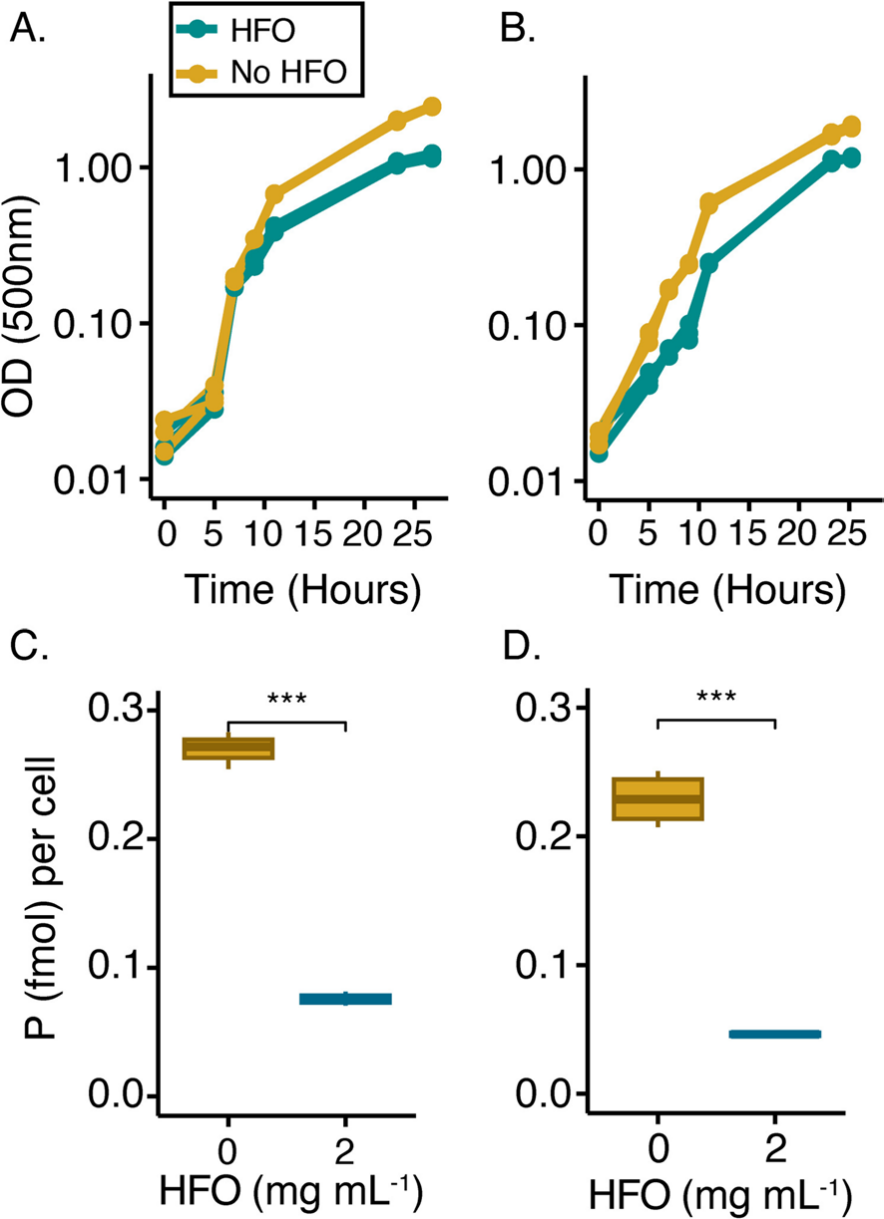
Phosphorus limitation of rhizosphere bacteria using HFO. *Pseudomonas* Root 71 (**A**, **C**) and *Rhizobium* Root 491 (**B**, **D**) grown with and without HFO (2 mg mL^−1^). **A**, **B** Growth measured at OD500. **C**, **D** Cellular phosphorus concentration for biological triplicates. A Bonferroni-corrected one-tailed *t*-test was used where *,**,*** indicate *p*-values less than 0.05, 0.01, 0.001, respectively. Box plot shows median and interquartile range

Next, we investigated whether the addition of HFO would also lead to phosphorus limitation in rhizosphere bacteria. We utilized two bacterial strains (*Rhizobium* Root 491 and *Pseudomonas* Root 71) that were previously isolated from *A. thaliana* roots as part of the *Arabidopsis* rhizosphere synthetic community (At-RSPHERE, Bai et al. 2015). To mimic phosphorus and HFO conditions that were effective for limiting plant phosphorus, both strains were grown in defined media containing 500 $\mu \mathrm{M}$ phosphorus with or without 2 mg HFO mL^−1^ (Fig. 3a, b). HFO was introduced in dialysis bags to contain the mineral and avoid interference with optical density measurements (see methods). For *Rhizobium* Root 491 and *Pseudomonas* Root 71, HFO addition led to decreased growth yields, dropping from OD_500_ = 1.87 and 2.46 in untreated controls to OD_500_ = 1.19 and 1.20 (respectively) in the prescence of HFO, indicating limitation (Fig. 3a, b). To verify that this reduction in yield was due to phosphorus limitation, bacterial cultures were filtered and cellular phosphorus concentration was measured with ICP-MS (Fig. 3c, d). For *Rhizobium* Root 491 and *Pseudomonas* Root 71*,* we found a significant (*p* < 0.001) reduction of 80% and 72% respectively in cellular phosphorus concentration in the HFO condition compared to the no HFO control (Fig. 3c, d), demonstrating that bacterial growth is limited for phosphorus in this system.

### Co-culture of *A. thaliana* and rhizosphere isolates

**Fig. 4:**
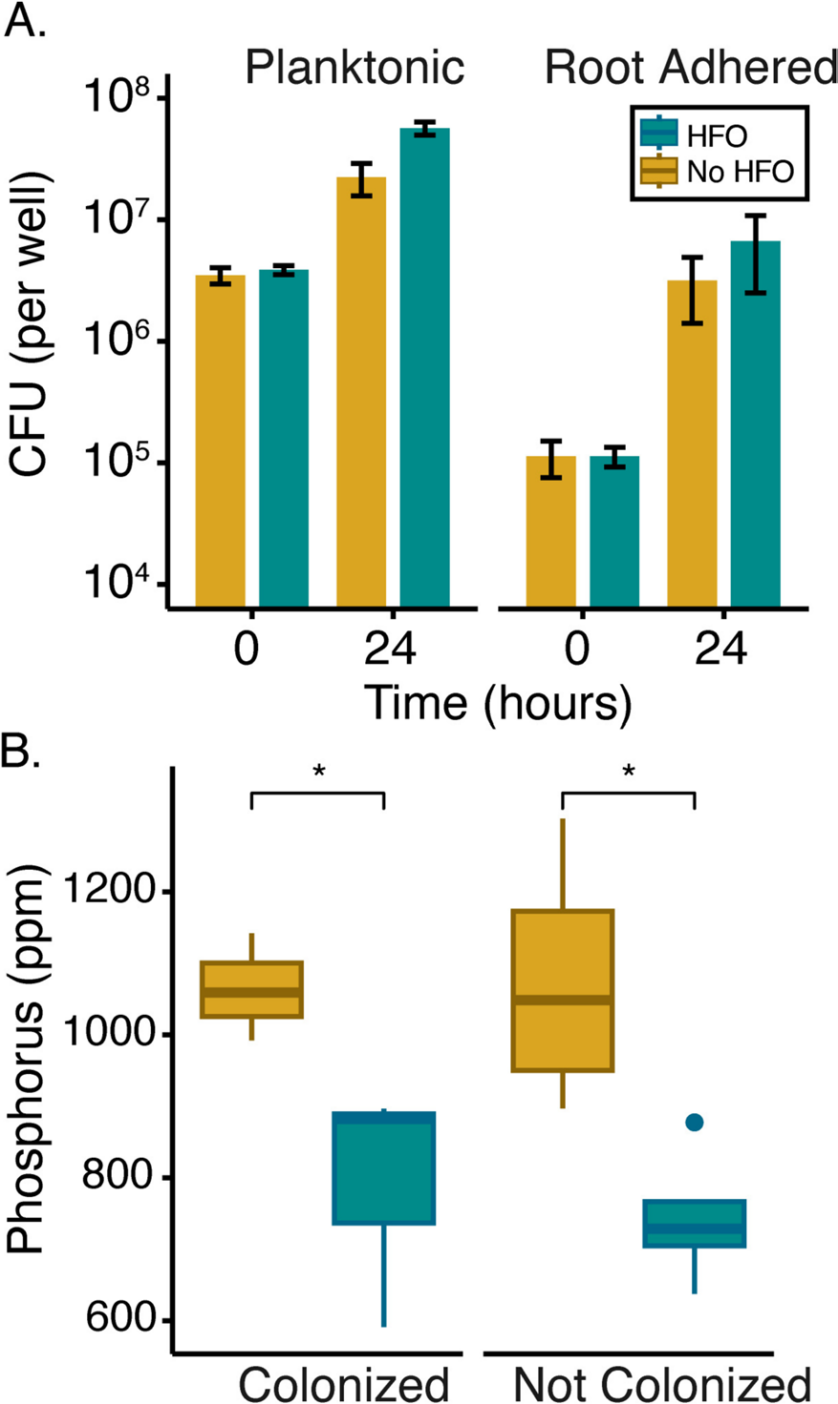
Plant microbe co-culture. *A. thaliana-Rhizobium* Root 491 co-cultures grown in 0.5 × MS media with (cyan) or without (yellow) 2 mg mL^−1^ HFO. **A** Growth of *Rhizobium* Root 491 in co-culture with or without *A. thaliana* for 24 h. Cell counts (per well) were determined with CFU (colony forming unit) counting. Error bars show the average of biological triplicates with ± 1 standard deviation. **B** ICP-MS measurements of *A. thaliana* P concentration (ppm). Three replicates were analyzed per condition. A Bonferroni-corrected one tailed t-test was used where *,**,*** indicate *p* less than 0.05, 0.01, 0.001, respectively. Box plot shows median and interquartile range

The ultimate aim for this hydroponic system is to develop a tool that allows for the co-culture of plants and microbes in soil-relevant conditions. To test this ability, we inoculated the roots of 20-day-old *A. thaliana* seedlings grown with or without HFO with *Rhizobium* Root 491. In natural root systems, one of the main drivers of plant–microbe interactions is the reliance of bacteria on plant photosynthates as a carbon source. We used bacterial reliance on plant carbon as a metric for assessing successful co-culture experiments. To test for robust root colonization by the inoculated *Rhizobium* Root 491 strain, the total cells in both free living (planktonic) and root adhered populations were quantified 24 h after inoculation. Colony forming unit counts for planktonic cells without HFO increased by a little more than an order of magnitude from 3.68 × 10^6^ to 3.95 × 10^7^ per well while root-adhered cells increased from 1.13 × 10^5^ to 5.52 × 10^6^ (Fig. 4a). Additionally, the presence of HFO (which in these experiments is added directly to the media, see methods) did not substantially alter the cell count of root adhered or planktonic bacteria (Fig. 4a), suggesting that bacterial colonization of iron particles is minimal.

As there was no carbon added to the 0.5 × MS media, the large increase in bacterial growth strongly implies reliance on plant produced carbon. However, as a further control for potential growth on background carbon, hydroponic media lacking a plant was inoculated separately with *Rhizobium* Root 491 and growth was quantified over 24 h. For total planktonic cell counts in these experiments, we observed a smaller increase from 4.18 × 10^6^ to 1.60 × 10^7^ (Supplementary Fig. 3a). A separate experiment conducted with the soil dwelling bacterium *Pseudomonas synxantha* grown in plant-free medium showed even more drastic results with a decrease in CFU counts over 24 h (Supplementary Fig. 3b). As a final test of this system, we compared phosphorus shoot concentrations in colonized vs uncolonized seedlings (Fig. 4b). In both cases, phosphorus shoot concentration was decreased when grown with HFO compared to the condition with no HFO (by ~ 25.8% in colonized plants and ~ 30.8% in uncolonized plants, Fig. 4b). Taken together, these results show that phosphorus limitation of *A. thaliana* is maintained in short-term co-culture with microbes and that bacteria are relying on plant photosynthates as their carbon source.

## Discussion

### Phosphorus adsorption and biological limitation in the presence of iron oxides

Our first experimental goal was to test the capacity of HFO to induce phosphorus stress and to develop standardized methods that can be applied in future studies. With a few exceptions, the results from our abiotic experiments were similar to those observed in previous studies on phosphate adsorption. Notably, in our experiments, mineral loading was 0.25 µmol phosphate per mg HFO (for 2 mg mL^−1^ treatments), which is far lower than previous studies where mineral loading is often in the range of 1–2 µmol per mg of mineral (Mallet et al. 2013; Wang et al. 2013; McRose and Newman 2021). However, the lower surface loading is consistent with a system where the HFO surface is not saturated with phosphate, which is desirable for scavenging soluble P, and would be expected as our incubations were conducted with 500 µM phosphate, whereas previous studies used millimolar phosphate solutions. We also observed mineral adsorption within minutes, which is consistent with previous studies. Despite this rapid adsorption, a 48-h adsorption period, achieved either during the plant etiolation phase or through pre-incubation before the addition of bacteria, is suggested as it is both experimentally convenient and appears sufficient for adsorption of dissolved phosphorus. It should be noted that our experiments were conducted at a relatively low pH of 5.5, where adsorption is expected to be the greatest; studies using a higher pH may require a higher mineral concentration for effective scavenging of phosphate.

Using these methods, our results clearly show we can limit both *A. thaliana* and rhizosphere bacteria for phosphorus through the addition of HFO (Figs. 2, 3). Our 0.5 × MS media has an N:P ratio of 60 (30 mM N to 500 µM P) and hence is phosphorus limited even in the absence of HFO. However, we observed progressive declines in tissue phosphorus concentration with increasing HFO (Fig. 2), demonstrating that mineral-adsorbed phosphorus is not available to plants. ICP-MS measurements of *A. thaliana* phosphorus shoot concentrations ranged from 1200 ppm without HFO additions to 325 ppm in treatments with 3 mg mL^−1^ HFO. These values fall at the lower end of previously reported phosphorus shoot concentrations for *A. thaliana* laboratory experiments conducted under varying phosphorus regimes, which range from 2500 to 300 ppm (Hanlon et al. 2018; Reis et al. 2020; Evert and Eichhorn 2013b, p. 29). Field surveys of plants across different soil types also report phosphorus tissue concentrations of a few thousand ppm (Han et al. 2005; Reich and Oleksyn 2004). Of note is the fact that these prior studies report concentrations normalized to fresh weight. Our values are normalized to dry weight and would be even lower if normalized to fresh weight. Unlike cellular P, chlorophyll concentration was stable across HFO treatments (Supplementary Fig. 4). This response is also expected for phosphorus limitation—although stress for this nutrient affects photosynthetic capacity, it typically does not result in chlorosis or measurable changes in bulk chlorophyll (Carstensen et al. 2018). The addition of HFO also led to differences in bacterial growth yields as well as cellular phosphorus quotas (Fig. 3). Both *Rhizobium* Root 491 and *Pseudomonas* Root 71 showed decreased P concentration in the presence of HFO: with *Rhizobium* Root 491 decreasing from 0.23 to 0.046 femtomole cell^−1^ and *Pseudomonas* Root 71 decreasing from 0.27 to 0.076 femtomole cell^−1^, on par with measured values for P-limited cells of similar sizes (Vrede et al. 2002; Chrzanowski and Grover 2008).

Previous studies of buffered phosphate supply systems opted to use aluminum oxides to avoid potential confounding interactions from iron (Hanlon et al. 2018). An especially promising outcome of our study is that our experimental techniques achieved phosphorus limitation without introducing iron limitation (Fig. 2). In sharp contrast to our phosphorus results, ICP-MS measurements of *A. thaliana* iron shoot concentration ranged from 250 to 750 ppm but did not show variation with HFO addition. This is expected for plants that are not limited for iron, where replete tissue concentration is often a few hundred ppm Fe (Gautam et al. 2022; Harbort et al. 2020; Cassin et al. 2009; Rajniak et al. 2018; Evert and Eichhorn 2013b, p. 29). Our relatively stable measurements of chlorophyll concentrations across treatments also support the conclusion that plants were not iron limited, as deficiency for this element typically leads to lower chlorophyll concentration (Eroglu et al. 2016). We also tested the use of the chelator EDTA to further buffer iron in the medium. While EDTA did appear to solubilize some mineral adsorbed P (as might be expected, see below), phosphorus limitation was still maintained, likely due to the large excess of Fe as compared to both EDTA and P. This result is a positive outcome for future studies interested in using HFO to sorb P while still ensuring replete Fe. Overall, while phosphorus stress responses in both plants and bacteria extend beyond simple elemental quotas our results provide a *prima facie* demonstration of P limitation using HFO.

### Utility for future investigations of phosphorus solubilization strategies

An experimental system with reduced complexity that mimics key features of the soil chemistry is important for studying phosphorus as many of the biological strategies to assuage P limitation occur outside the cell and are designed to modify environmental conditions. Under low phosphorus, both plants and microbes release small molecules and enzymes that solubilize phosphorus through a variety of mechanisms, including acid-catalyzed dissolution, metal chelation, enzymatic degradation, and redox processes (Hayat et al. 2010; Pan and Cai 2023; Goldstein 1986; Gerretsen 1948; Sperber 1957; Ferrol et al. 2019). Some of these strategies, such as phosphatase production, are aimed at accessing organic P and will not be relevant to our experimental system, but others explicitly require study in systems with HFO-adsorbed phosphate. For example, the production of secondary metabolites such as siderophores [which bind Fe(III)] and reductants [which can reduce Fe(III) to Fe(II)] are biological strategies that have received a great deal of attention in the context of iron limitation. Many studies have successfully used iron minerals to document the effects of these molecules on mineral dissolution and iron-limited plant and microbial growth (Rajniak et al. 2018; Hernandez et al. 2004; Cheah et al. 2003; Akafia et al. 2014; Reichard et al. 2007; Baune et al. 2020). However, there is emerging evidence that these same mechanisms are also likely to increase bioavailability of phosphorus adsorbed to iron oxides. Indeed, reductive dissolution of phosphated iron minerals is well documented (Stumm and Sulzberger 1992; Van Cappellen and Ingall 1996), and we recently demonstrated that many bacteria produce reductants in response to phosphorus stress and that these metabolites can enhance phosphorus bioavailability (McRose and Newman 2021; Giacalone et al. 2024). The ability to conduct phosphorus limitation experiments with HFO holds special utility for continued study of these types of phosphorus solubilization strategies that rely on the reduction or chelation of iron.

A second reason that more realistic experimental systems for investigations of soils are needed is that the distribution of soil nutrients is patchy, and well mixed systems fail to capture phenotypic responses to uneven nutrient distributions. This facet of soils has been a persistent challenge for researchers but is increasingly embraced as a core feature of the soil environment. While our work does not directly address the physical complexity of soils, it does provide a tool to enable more realistic studies of the chemical aspects of soil nutrients that might be fruitfully paired with existing methods to mimic soil physical structure. The importance of soil heterogeneity is most obviously important for studies of plants, where the alteration of root growth and morphology can help to increase encounter rates with soils nutrients such as adsorbed phosphate (Lynch et al. 2021; Hanlon et al. 2018; Evert and Eichhorn 2013a). The need to access unavailable phosphorus also underpins the cooperative relationships between plants and mycorrhizal fungi as well as soil-dwelling bacteria (Castrillo et al. 2017; Becquer et al. 2014), and these interactions are shaped in part by the inherent chemical and physical heterogeneity of the soil system.

In addition to showing that HFO can be used to induce limitation in *A. thaliana* and root-associated bacteria, we provide a proof-of-concept demonstration that HFO can be used for bacterial root colonization experiments while still allowing bacteria to be recovered and counted. While the nature of the interaction between plants and root bacteria is more complex than simple carbon exchange, our finding of bacterial reliance on plant carbon as well as bacterial growth on roots offers a necessary first demonstration of the utility of this system. Our work also shows that short term colonization with bacteria does not affect plant phosphorus concentration. Phosphated HFO provides a tool for future work that might investigate the effects of longer-term bacterial colonization on plant access to phosphorus. Given the recent advances in techniques to mimic soil structure, phosphated HFO might also easily be combined with synthetic soil scaffolds, beads, or lithography-based tools (Sharma et al. 2020; Boiteau et al. 2021; Smercina et al. 2022; Bhattacharjee et al. 2024). Such studies would require further optimization but could be a promising means to investigate both chemical and physical soil heterogeneity, probing features of the system such as root morphology and carbon exudation as well as spatially structured bacterial growth at the plant root. These types of investigations will offer powerful insights into the convergence of plant and microbial genotypes, phenotypes, and metabolites that enable the extraction of this essential nutrient from the environment.

## Conclusion

We present a gnotobiotic hydroponic method to co-culture plants and rhizosphere microbes utilizing iron oxides to mimic the chemical properties of natural soils. Our results demonstrate that we can limit both plants and microbes for phosphorus through mineral adsorption without inducing limitation for iron. We also show that this system is a viable method for co-culture experiments as hydroponically grown plants were successfully colonized with rhizosphere bacteria that relied on plant photosynthates as their sole carbon source. This technique can be used to further explore the physiology of plants and microbes and the ways they work together to tolerate nutrient limiting conditions across managed and natural systems.

## Supplementary Information

Below is the link to the electronic supplementary material.

Supplementary file1 (PDF 89716 KB)

## Data Availability

Data is provided within the manuscript or supplementary information files.
