# Supplementary material for: Gnotobiotic growth and phosphorus limitation of Arabidopsis thaliana and co-occurring microbes on phosphated iron oxides
Source: Biometals. 2025 Nov 27;39(1):359–73. doi: 10.1007/s10534-025-00767-6 (PMC12852198; doi:10.1007/s10534-025-00767-6)
Supplement: Supplementary file 1 — Supplementary file1 (PDF 89716 KB) [file 10534_2025_767_MOESM1_ESM.pdf]

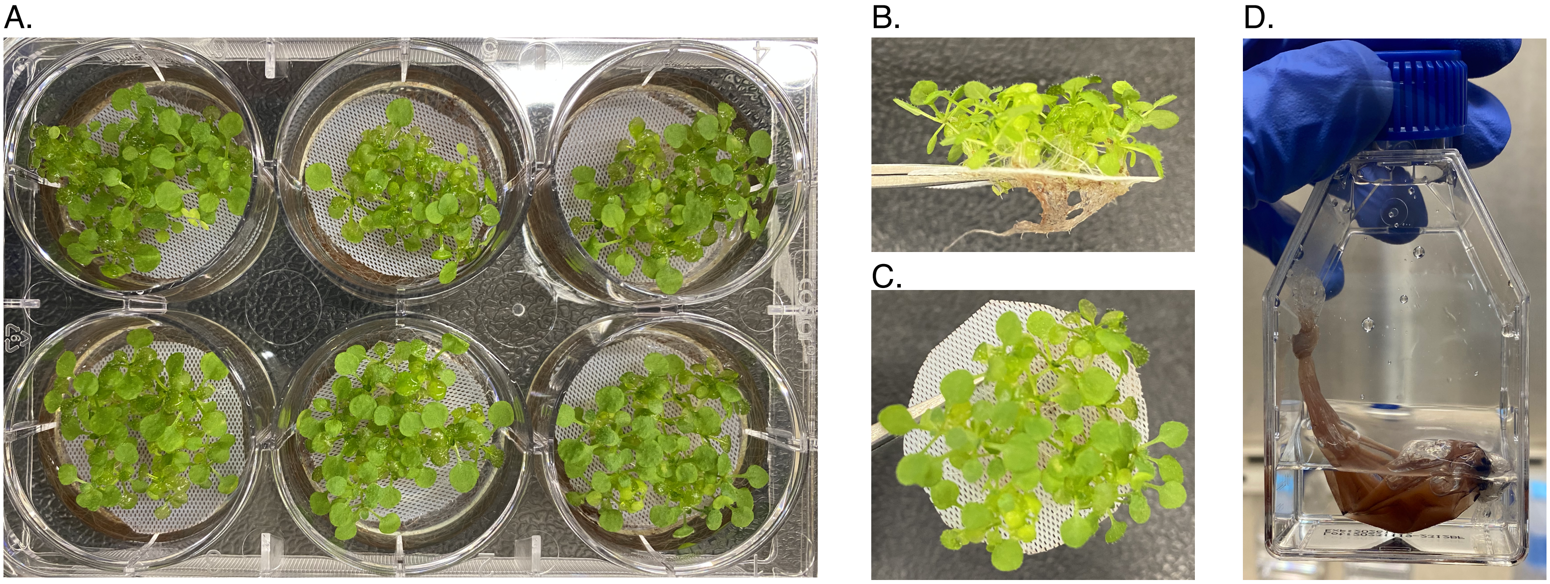

Figure S1. Experimental growth setup for plants and bacteria. Top (A,C) and side (B) view of hydroponically grown plants. D: HFO dialysis bag used for bacterial culture.

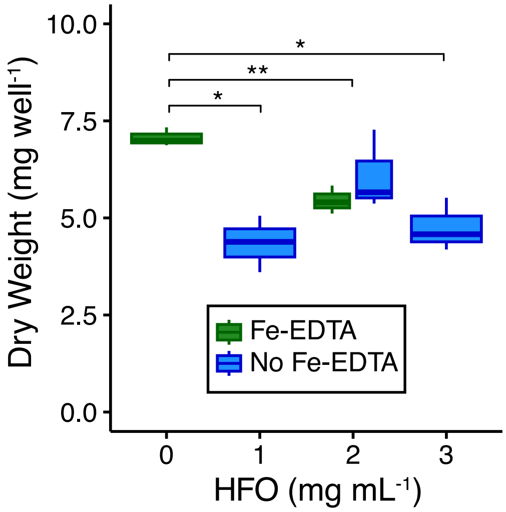

Figure S2. Plant dry weight across HFO concentrations. *A. thaliana* was grown hydroponically in 0.5x MS media containing 500 μM phosphorus and various concentrations of HFO. Three replicate wells were analyzed per condition. Fe-EDTA (50 μM $FeCl_3$ and 100 μM EDTA) was included for the no HFO condition and for one 2 mg HFO mL^{-1} condition (green). Treatments without Fe-EDTA are indicated in blue. Bonferroni-corrected two-tailed t-test was used where *,**,*** indicate p-values less than 0.05, 0.01, 0.001, respectively. No significance is indicated by ns. Box plot shows median and interquartile range.

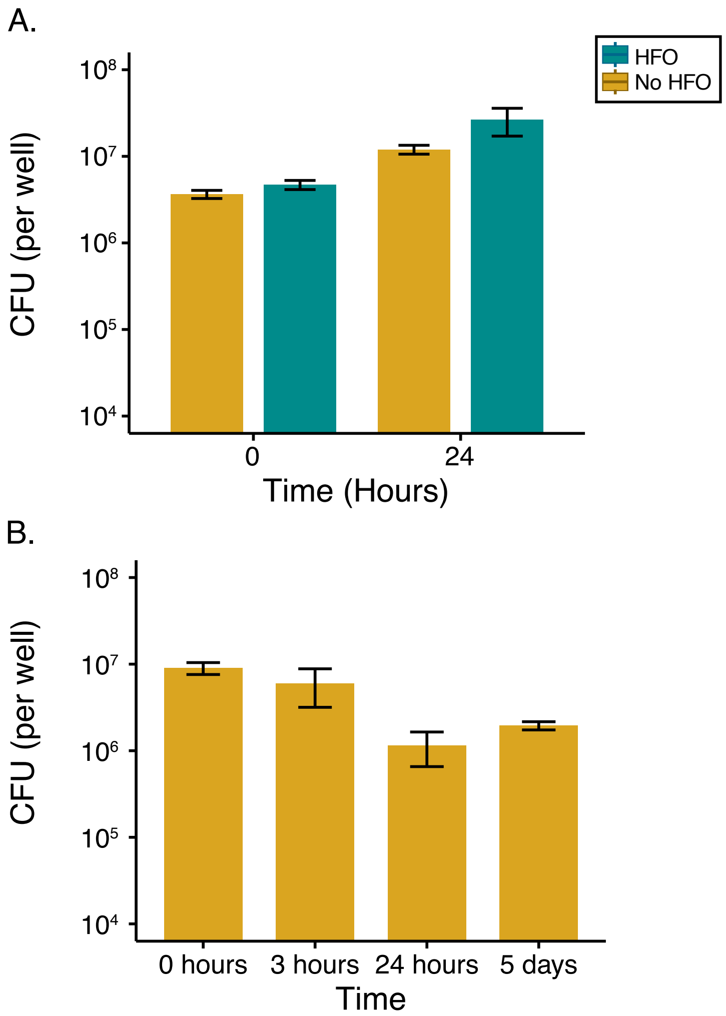

Figure S3. Bacterial growth in the absence of plants is minimal. *Rhizobium* Root 491 (A) or *Pseudomonas synxantha* (B) was cultured in 0.5x MS media without *A. thaliana*. Cell count was determined with CFU (colony forming unit) counting. Error bars show the average of biological triplicates with ± 1 standard deviation.

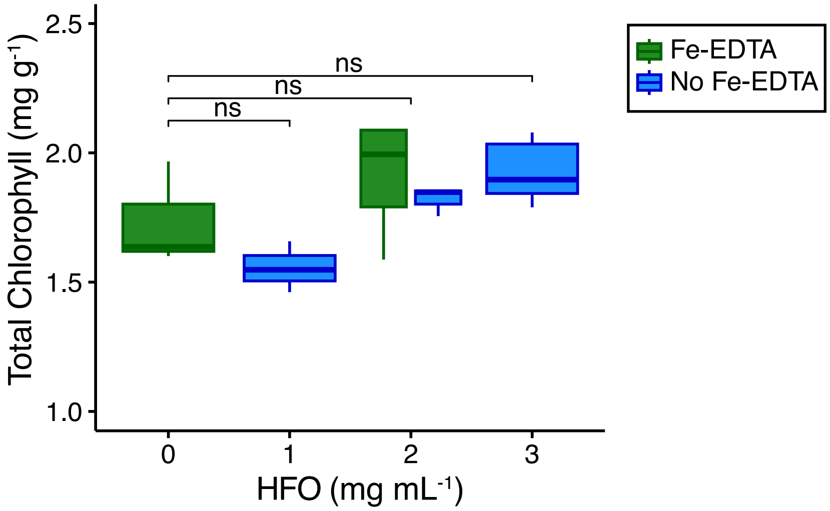

Figure S4. Total chlorophyll is stable across HFO concentrations. *A. thaliana* was grown hydroponically in 0.5x MS media containing 500 µM phosphorus and various concentrations of HFO. Chlorophyll (mg mL^{-1}) was extracted with ethanol and measured via absorbance. Three biological replicates were analyzed per condition. Fe-EDTA (50 µM $FeCl_3$ and 100 µM EDTA) was included for the no HFO condition and for one 2 mg HFO mL^{-1} condition (green). Treatments without Fe-EDTA are indicated in blue. Bonferroni-corrected two-tailed t-test was used where *,**,*** indicate p-values less than 0.05, 0.01, 0.001, respectively. No significance is indicated by ns. Box plot shows median and interquartile range.
